# Phylogenetic Patterns of Geographical and Ecological Diversification in the Subgenus *Drosophila*

**DOI:** 10.1371/journal.pone.0049552

**Published:** 2012-11-12

**Authors:** Ramiro Morales-Hojas, Jorge Vieira

**Affiliations:** Molecular Evolution Lab, Instituto de Biologia Molecular e Celular, Universidade do Porto, Porto, Portugal; North Carolina State University, United States of America

## Abstract

Colonisation of new geographic regions and/or of new ecological resources can result in rapid species diversification into the new ecological niches available. Members of the subgenus *Drosophila* are distributed across the globe and show a large diversity of ecological niches. Furthermore, taxonomic classification of *Drosophila* includes the rank radiation, which refers to closely related species groups. Nevertheless, it has never been tested if these taxonomic radiations correspond to evolutionary radiations. Here we present a study of the patterns of diversification of *Drosophila* to test for increased diversification rates in relation to the geographic and ecological diversification processes. For this, we have estimated and dated a phylogeny of 218 species belonging to the major species groups of the subgenus. The obtained phylogenies are largely consistent with previous studies and indicate that the major groups appeared during the Oligocene/Miocene transition or early Miocene, characterized by a trend of climate warming with brief periods of glaciation. Ancestral reconstruction of geographic ranges and ecological resource use suggest at least two dispersals to the Neotropics from the ancestral Asiatic tropical disribution, and several transitions to specialized ecological resource use (mycophagous and cactophilic). Colonisation of new geographic regions and/or of new ecological resources can result in rapid species diversification into the new ecological niches available. However, diversification analyses show no significant support for adaptive radiations as a result of geographic dispersal or ecological resource shift. Also, cactophily has not resulted in an increase in the diversification rate of the *repleta* and related groups. It is thus concluded that the taxonomic radiations do not correspond to adaptive radiations.

## Introduction

The large diversity of life forms that we see today is the result of different biological processes, one of which is adaptive radiation [1], [2], [3]. Adaptive radiations refer to lineages that exhibit a diversification of species into different ecological niches. This process is generally triggered by ecological opportunity, in which an underutilized environment becomes available to an ancestral species allowing a rapid species diversification into the new ecological niches available. The new environment can be colonized either by the evolution of a key innovation, the dispersal into a new geographic area or by the extinction of antagonist species [4], [5]. The study of the rates of diversification across phylogenetic lineages can be used to identify adaptive radiations and provides valuable information about the processes that underlie the origin of biological diversity [3]. A pattern that is generally considered to be the result of an adaptive radiation is when there is a rapid origin of species that adapt to a diversity of ecological niches followed by a slow down of the diversification rate through time as the new niches become occupied [2], [6]. This is a common pattern observed in many taxonomic groups (e.g. [7], [8], [9], [10]).

The genus *Drosophila* is a large, diverse and widely distributed group of organisms [11]. Its taxonomy is relatively well established and, while the phylogeny, ecology and distribution for some species are not well characterized, there is broad information for most of the species groups [12], [13]. There is some difficulty in resolving the phylogenetic relationships between the main groups of *Drosophila*, which has been attributed to the rapid divergence of these lineages as the descendants adapted to a variety of ecological resources [12]. This pattern, if confirmed, could indicate that the *Drosophila* lineage is the result of an adaptive radiation.

The genus *Drosophila* is paraphyletic as several other genera are included within the phylogeny of the *Drosophila* [12]. Ten subgenera are presently recognized within the genus *Drosophila*, of which the *Sophophora* and *Drosophila* are the major ones [14]; these are further taxonomically subdivided into radiations and species groups. It should be noted that the term radiation refers to a taxonomic rank that comprises several closely related species groups, and should not be confused with an adaptive radiation. Furthermore, it has never been tested whether these taxonomic radiations correspond to adaptive radiations. Of the two main subgenera, *Drosophila* is the largest one. It has a wide distribution and some of its members show interesting ecological niches such as fungi and cacti. It comprises three major lineages: 1) the *funebris* species group; 2) the *virilis*-*repleta* radiation; and 3) the *immigrans*-*tripunctata* radiation, which following Remsen and O’Grady [15] excludes the genus *Hirtodrosophila* in contrast to the *immigrans-Hirtodrosophila* radiation of Throckmorton [11]. The phylogenetic position of the *funebris* group is not well resolved, and some studies suggest that it is part of the *immigrans-tripunctata* radiation [15], [16]. It should be noted that the subgenus *Drosophila* is paraphyletic and includes the Hawaiian Drosophilidae (Hawaiian *Drosophila* + genus *Scaptomyza*), which form a monophyletic, sister group to the *virilis-repleta* radiation [15], [17] or to the *virilis*, *robusta*, *melanica* clade within the *virilis-repleta* radiation [18]. This Hawaiian lineage comprises approximately 1000 species and it forms an adaptive radiation of its own, with a large diversity of forms and ecological niches [19].

Based on biogeographic data, the origin of the *virilis-repleta* radiation has been placed in the Old World tropics, most likely in the Asiatic tropical regions [11]; from this ancestor two lineages evolved leading to the Old World tropics (e.g. the *polychaeta* group) and temperate species groups (e.g. *virilis*, *robusta* and *melanica* species groups). A Neotropical radiation, which comprises the *repleta*, *canalinea*, *mesophragmatica*, *dreyfusi*, *annulimana* and *nannoptera* species groups, evolved from a third lineage of the Asiatic tropical ancestor. The origin of the *immigrans-tripunctata* radiation has also been placed in the Old World tropics, from where two lineages arose, the Asiatic *immigrans* group and the New World *tripunctata* radiation that comprises the *tripunctata* and closely related species group [11]. From an ecological point of view, the members of the subgenus *Drosophila* occupy a wide variety of niches, from sap feeders (e.g. *robusta*, *melanica* and *virilis*) to cactophilic species (e.g. *repleta*), mycophagous (e.g. *quinaria*) and flower feeders (e.g. *tripunctata*) [13], [14], although many of the species are generalists and can exploit different resources. Cactophily is not observed in any species outside the lineage including the *repleta*, *nannoptera* and *mesophragmatica* species groups. As the ability to exploit cacti as an ecological resource implies acquiring the capacity to degrade an array of toxic compounds that are produced in rotting cacti material [20], [21], cactophily can be considered an apomorphy of this Neotropical lineage.

Given the widespread geographic distribution of the different species groups and the variety of the ecological resources they exploit, the subgenus *Drosophila* could represent an adaptive radiation. In order to test for this hypothesis we have estimated a time-calibrated phylogeny of 218 species representing the main species groups of the subgenus *Drosophila*. This phylogenetic analysis is the first attempt to date the divergence events of the main lineages of this group of organisms using a relaxed molecular clock approach. Therefore, the results of the present study will be relevant for many research areas because the subgenus *Drosophila* includes some of the best-studied model organisms in ecological and evolutionary research [22], [23], [24], [25], [26], [27], [28]. Based on the obtained phylogeny, and taking into account topological and dating uncertainties, we have reconstructed the ancestral states for the species’ geographic distribution and ecological resource use. We have also investigated the patterns of diversification of those lineages that dispersed into the Neotropic and acquired the capacity to exploit new resources.

## Materials and Methods

### Samples and Gene Sequences

The phylogeny of the subgenus *Drosophila* was reconstructed using sequences for the nuclear *Adh* and the mitochondrial *ND2* and *COI* for 218 species representing all the major lineages of the subgenus (table S1; sequences were obtained from GenBank with exception of the *Adh* sequences of some species of the *virilis* group, obtained by us [29]). More than 70% of the species included in the analyses have sequence data for at least two of the genes. While some of the taxa had data for just one gene, it has been shown that highly incomplete taxa can still be accurately placed in the phylogeny if there are overlapping characters [30]. As the DNA alignment included non-overlapping characters, analyses were also run with 153 species for which there was sequence data for 2–3 genes. This controlled for any potential bias introduced in the phylogenies by non-overlapping characters.

Alignment of gene sequences was done with Clustal X v2.0 [31] with minor adjustments by eye based on the amino-acid translation of the cds to avoid alignment of non-orthologous nucleotides and distortion of the ORF. Alignment editing and coding sequence translation was done in Se-Al v2.0a11 [Rambaut (1996) http://tree.bio.ed.ac.uk/software/seal/].

### Phylogenetic Analyses

Phylogenetic reconstruction was performed using Bayesian Inference (BI) and Maximum Likelihood (ML) as optimality criteria. For the BI approach, phylogenetic relationships between the species were estimated at the same time as the estimation of the divergence times with BEAST version 1.7.2 [32] run in CIPRES Science Gateway [33]. The divergence times were estimated under the uncorrelated relaxed-clock tree model [34]. Runs were performed allowing for different substitution rates and clocks at nuclear and mitochondrial genes. The model of evolution used for each data partition was the GTR+I+G. Runs were performed with two different matrices: 1) all 218 species, which included non-overlapping characters; and, 2) alignment of 153 species with overlapping sequences (2–3 genes). Also, two calibration schemes were used in these analyses. First, the calibration of the tree was done using dates of divergence obtained from the literature for 9 nodes: 1) *antopocerus-modified tarsi* species groups, mean = 9 Mya±1.0 (standard deviation) [35]; 2) *haleakalae* species group, mean = 10 Mya±1.0 (stdev) [35]; 3) *modified mouthparts* group, mean = 16 Mya±1.0 (stdev) [35]; 4) *picturewing*-*nudidrosophila* groups, mean = 15 Mya±1.0 (stdev) [35]; 5) *D. picticornis* - *planitibia* group, mean = 4.6 Mya±0.3 (stdev) [36], [37]; 6) *virilis* group, mean = 9.4 Mya±0.6 (stdev) [29]; 7) *virilis* subgroup, mean = 4.05 Mya±0.6 (stdev) [29]; 8) *montana* subgroup, mean = 4.9 Mya±0.5 (stdev) [29]; and 9) *D. mojavensis - D. arizonae*, mean = 2.4 Mya±0.3 (stdev) [38]. Calibrating times were introduced in the analysis as priors with a normal distribution; the standard deviation specified for each calibration point was chosen to include the confidence limits reported in the respective studies. As some of these calibration points are approximate estimates and had no confidence limits associated to them, analyses were also run using only 5 calibration points based on geological and climatic data and that had associated intervals: 1) *D. picticornis* - *planitibia* group, mean = 4.6 Mya±0.3 (stdev) [36], [37]; 2) *virilis* group, mean = 9.4 Mya±0.6 (stdev) [29]; 3) *virilis* subgroup, mean = 4.05 Mya±0.6 (stdev) [29]; 4) *montana* subgroup, mean = 4.9 Mya±0.5 (stdev) [29]; and 5) *D. mojavensis - D. arizonae*, mean = 2.4 Mya±0.3 (stdev) [38]. The divergence between *D. picticornis* and other species from the *planitibia* subgroup has been estimated to have occurred 5.1 mya based on the oldest surface rock of the island of Kauai [36], [37]. The mean value and associated standard deviation introduced as normal prior was chosen to incorporate the 1 my time span (5.1–4.1 mya) described to elapse between the pre-shield and shield stages according to the models of volcano growth. As outgroups we used *D. melanogaster*, *D. yakuba*, *D. ananassae*, *D. pseudoobscura* and *D. willistoni*. The analyses with 218 spp. and 9 calibration points were run for 200×10^6^ generations, with sampling every 10000^th^ generations. The first 5000 trees were discarded as burn-in and the remaining 15001 phylogenies were summarized using maximum clade credibility. The other three analyses (218 spp.-5 calibration points, 153 spp.-9 and -5 calibration points) were run for 100×10^6^ generations, sampling every 10000^th^ generations. The burn-in was set to 10%. In order to confirm that the MCMC had run long enough to get valid estimates and establish the burn-in level, results were analysed with TRACER [39] and the effective sample size (ESS) confirmed to be greater than 200.

Maximum Likelihood (ML) analyses were run using RAxML 7.2.8 [40], [41] in CIPRES Science Gateway [33]. The analysis was performed with a partitioned dataset (one model for each gene *Adh*, *ND2* and *COI*) and 1000 rapid bootstrap inferences were executed with a thorough ML search thereafter. To estimate clade support, 350 bootstrap replicates were performed.

### Ancestral State Reconstruction

Two different approaches were used for the ancestral character state reconstruction of the geographic distribution and ecological resource use. First it was used the likelihood method implemented in Mesquite [42]. Character mapping was done on the summarized BI chronogram (218 spp. +9 calibration points) with the Hawaiian Drosophilidae clade pruned. Being a monophyletic, derived lineage its removal will not affect the assignment of ancestral states for the main lineages of the study. Furthermore, this lineage has been comprehensively investigated in a recent study [19].

In order to account for phylogenetic uncertainty, the Bayesian ancestral state reconstruction implemented in SIMMAP v1.5 [43], [44] was used. This method estimates the marginal posterior probabilities of each possible character state at the internal nodes of a sample of phylogenies. To account for topological uncertainty we used a random sub-sample of 100 trees from the posterior distribution of phylogenies obtained with BEAST (218 spp, 9 calibrations). The parameters for the prior distributions of the models implemented in the analyses were estimated using an MCMC approach as described in the SIMMAP webpage.

The number of categories introduced in the ancestral reconstruction was restricted by the limitation of SIMMAP 1.5, which accepts a maximum of 7 character states. The biogeographic categories used in the analyses were based on the regions of TaxoDros (www.taxodros.uzh.ch): 0 - cosmopolitan (when one species is found in more than one region); 1 - European; 2 - African; 3 - North American; 4 - Neotropical; 5 - Asian; and 6 - Australia + Oceania. We are aware that the records for some species from the database can be dubious, nevertheless, as we are assessing general trends for a rather large lineage we are confident that incorrect records for a small proportion of species will not bias the results or their interpretation. The ecological resources included were those natural substrates from where *Drosophila* adults and larvae have been collected. Following Throckmorton [11] and Markow and O’Grady [13] with additional information obtained from the literature (table S2 and references therein), the following categories were used: 0 - generalist (species that use more than one type of substrate except cacti); 1 - mycophagous; 2 - frugivorous; 3 - sap feeders; 4 - cactophilic; and 5 - generalist + cactophilic (those species that can use any part of any plant including cacti).

### Analyses of Diversification

Analyses of diversification were run with the phylogenetic results obtained with 218 spp. and 9 calibration points, and with 153 spp. and 5 calibration nodes to test for potential bias as result of the different divergence times obtained with the different calibration points.

The constant-rate (CR) test [45] was used to examine the departure of the lineage accumulation from the null hypothesis of a constant rate of diversification. The CR test evaluates the relative position of nodes in the phylogeny against the expected under a CR model of diversification using the γ statistic, calculated with LASER [46] in R. Negative values of γ indicate that the nodes are closer to the root than expected, signifying a deceleration in the rate of diversification; positive values indicate a bias towards the tips of the tree and denote acceleration in the diversification rate towards the present. The γ-statistic was computed for 1000 posterior distribution trees from each of the two BEAST analyses to control for the uncertainty in topology and branching times. Incomplete taxon sampling can bias the CR test and, in order to correct for this effect we adjusted the critical value using the MCCR test [45] implemented in LASER. The total number of species in the lineages analysed was obtained from Markow and O’Grady [14] and the TaxoDros database (Table 1). Number of Monte Carlo simulations run was 5000. To test the significance of the empirical γ value distribution estimated for the 1000 posterior trees, the average and median values of γ from the 1000 posterior trees were compared to the critical γ value obtained from the null distribution. A second source of bias in the γ-statistic comes from the violation of the random sampling assumption. It has been recently shown that non-random taxonomic sampling inflates the type-I error of the CR and MCCR tests [47]. The degree of bias introduced in the analysis by non-random sampling is here evaluated using the scaling parameter α as described by Brock et al. [47].

**Table 1:** Summary data for the species groups of the *Drosophila* subgenus.

Species group	Number spp.	Missing spp.
*angor*	7	0
*annulimana*	16	14
*calloptera*	8	5
*canalinea*	14	12
*cardini*	16	9
*clefta*	3	0
*dreyfusi*	9	8
*funebris*	7	6
*guarani*	16	11
*immigrans*	104	84
*macroptera*	5	4
*melanica*	13	4
*mesophragamatica*	13	7
*nannoptera*	5	2
*pallidipennis*	1	0
*polychaeta*	10	3
*quadrisetata*	12	2
*quinaria*	33	25
*repleta*	101	57
*robusta*	16	2
*sticta*	1	0
*testacea*	4	3
*tripunctata*	79	57
*virilis*	13	1
Hawaiian *Drosophila*	1000	972

Number spp. is the extant number of species, missing spp. is the number of species listed in TaxoDros and Markow and O’Grady [14] not represented in the phylogeny.

The temporal method BDL implemented in LASER was used to test for departure from constant rate and to detect rate shifts in the presence of extinction [48]. Rate-constant diversification models (RC) fitted to the data were pureBirth and birth-death models. The rate-variable (RV) models tested were the density-dependent models with exponential and logistic variants (DDX and DDL) and the yule2rate and yule3rate models, which allow for two and three different rates of speciation across the phylogeny, respectively. The models were fitted to the branching times of the maximum clade credibility (MCC) trees obtained with BEAST and shifts were only allowed at the branching times. The significance of the change in the Akaike Information Criterion (ΔAIC) scores between the RC and RV models was tested fitting the models to the branching times of a simulated sample of 5000 trees. In order to account for incomplete sampling, the trees were simulated to have the total number of species as the lineages analysed and were then pruned to contain the same number of tips as our phylogeny.

In order to detect exceptionally radiating lineages within the subgenus *Drosophila* we used MEDUSA [49] implemented in Geiger [50]. MEDUSA tests among-clade variation in rates of speciation (and not rate variation through time as the tests above) by combining phylogenetic information about the timing of splits with taxonomic richness data. The advantage of this method is that it accommodates incomplete sampling by using taxonomic richness information. MEDUSA was run with different backbone phylogenies containing 13 tips corresponding to the main monophyletic lineages of the phylogeny and combined with a species richness table (Table 1). The backbone phylogenies used were obtained by pruning the MCC trees from the 218 spp. with 9 calibrations and 5 calibrations analyses, to test for possible biases introduced by the different topologies and divergence dates. Those species groups that were paraphyletic, were combined into a single tip thus, the *robusta* clade was considered to have 44 spp. including the *robusta*, *melanica*, *quadrisetata* and *clefta* species groups, and the *tripunctata* clade (170 spp.) included the *tripunctata*, *sticta*, *pallidipennis*, *cardini*, *guarani*, *testacea*, *calloptera*, *funebris*, *quinaria* and *macroptera*. Species richness was taken from TaxoDros and Markow and O’Grady [14].

### Test of Macroevolutionary Hypothesis

Bursts of diversification can be the result of the invasion of previously unoccupied ecological niches. Whether adaptation to cacti in the *repleta* and closely related species groups has allowed an increased rate of diversification in this group has been tested with BiSSE [51], implemented in Mesquite [42]. BiSSE uses a likelihood-based approach to test the association of a discrete character, in the present case being cactophilic or not, with the rate of diversification of different lineages of a phylogeny. The likelihoods of our empirical data (summarized chronogram of the *Drosophila* subgenus and character states) were estimated under two models, unconstrained and constrained, using BiSSE. The unconstrained model had all parameters (λ, μ and q) free to vary while the constrained model was forced to have the same speciation rate for both character states (λ_0_ = λ_1_). The statistical significance of the log-likelihoods difference was tested with a likelihood ratio test assuming a χ^2^ distribution with one degree of freedom.

## Results

### Phylogeny of the Subgenus *Drosophila*

**Figure 1:**
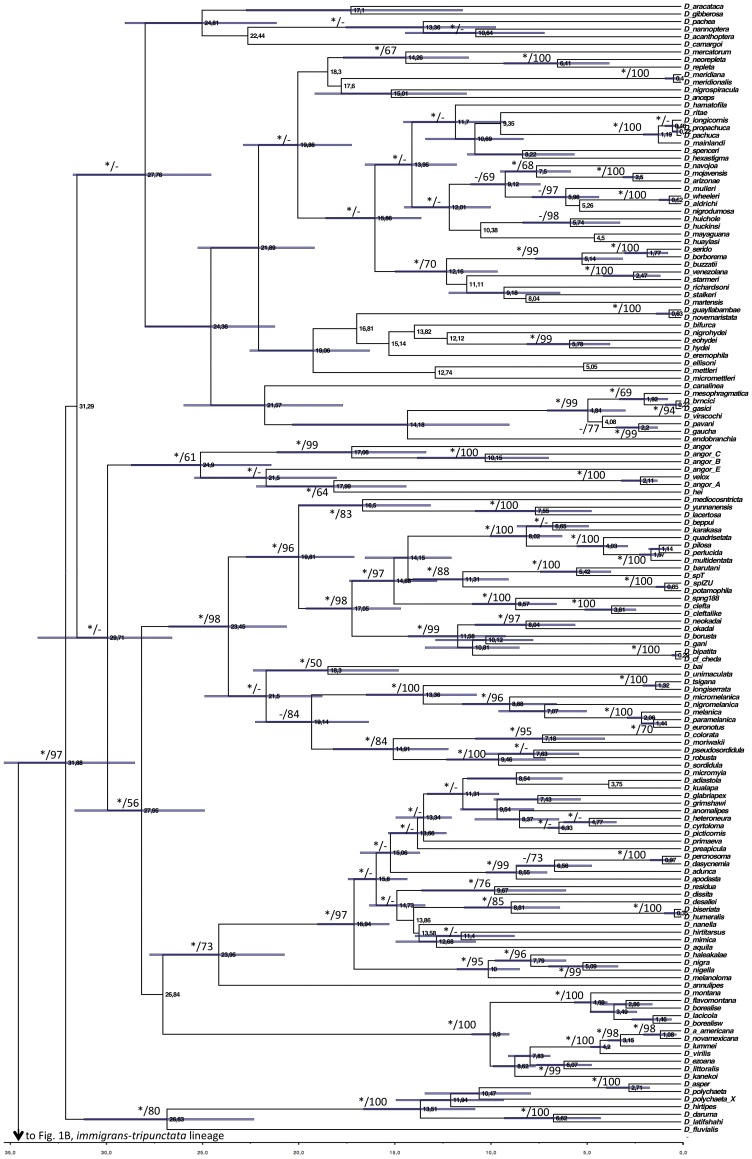
Summarized chronogram for the subgenus *Drosophila* obtained with BEAST using 218 species and 9 calibration points. Numbers on nodes indicate ages for the corresponding nodes; asterisks on branches indicate BPP ≥ 90% and numbers are bootstrap support values. Bars represent the 95% highest posterior density (HPD) interval for the divergence times.

**Figure 2:**
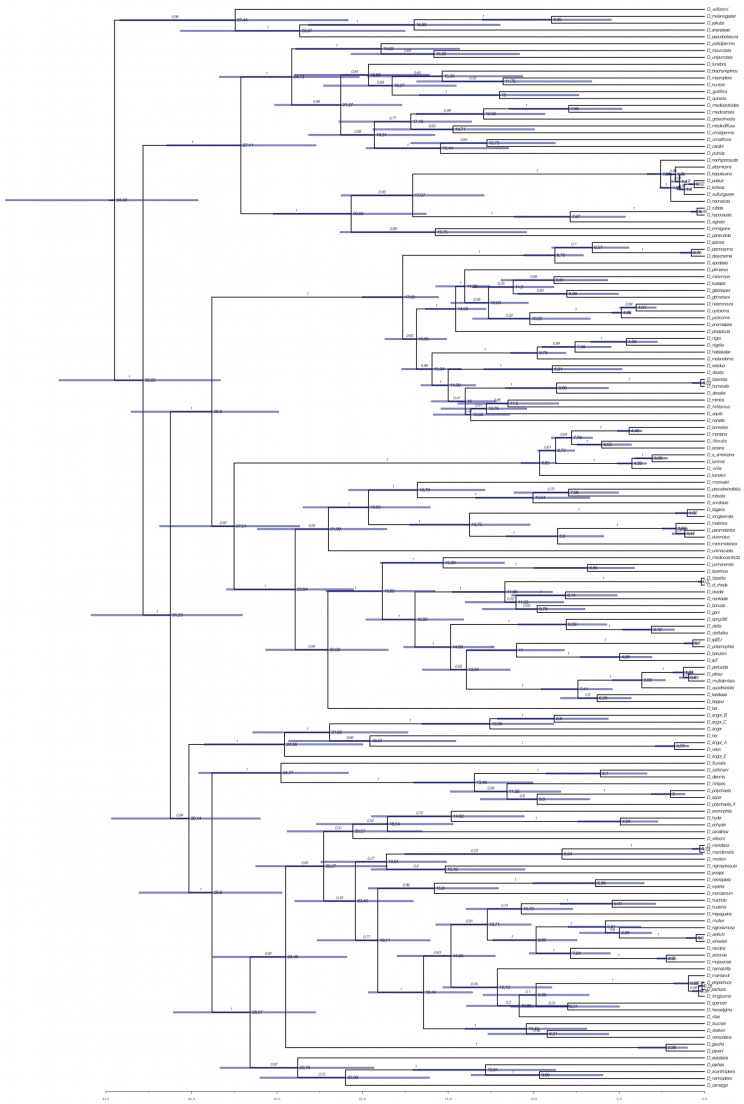
Summarized chronogram for the subgenus *Drosophila* obtained with BEAST using 153 species and 9 calibration points. Numbers on nodes indicate ages for the corresponding nodes; numbers on branches indicate BPP values. Bars represent the 95% highest posterior density (HPD) interval for the divergence times.

**Figure 3:**
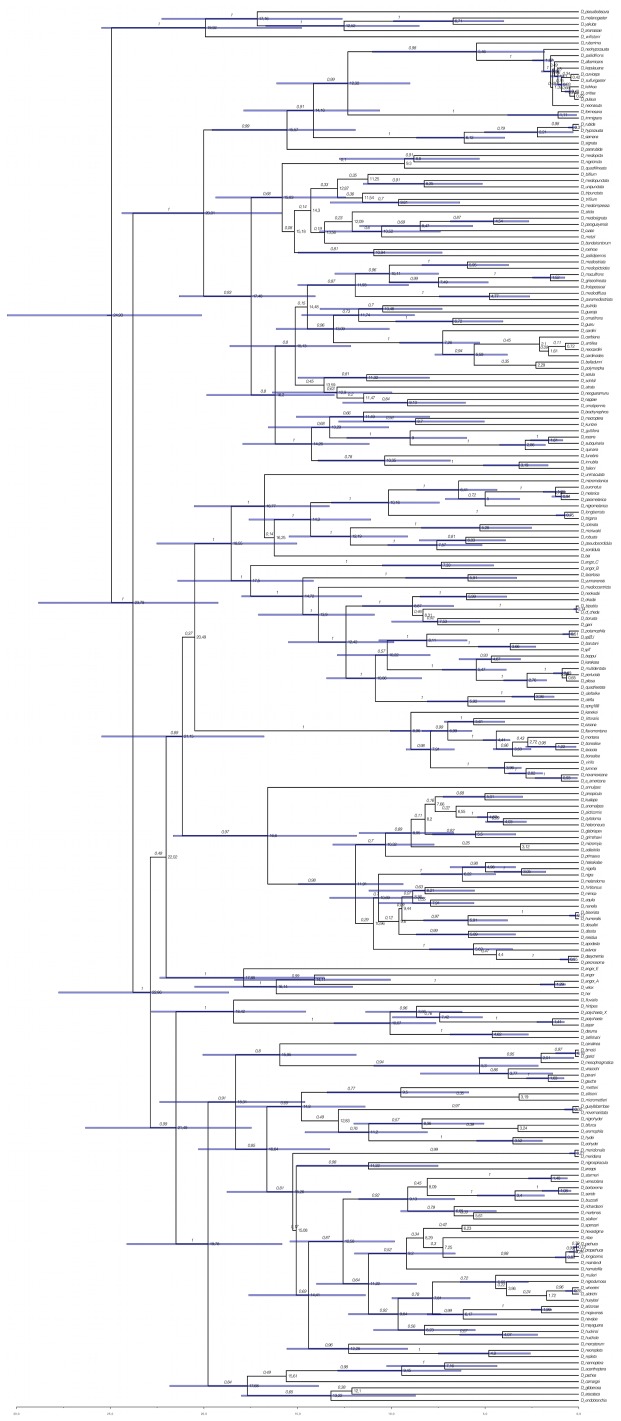
Summarized chronogram for the subgenus *Drosophila* obtained with BEAST using 218 species and 5 calibration points. Numbers on nodes indicate ages for the corresponding nodes; numbers on branches indicate BPP values. Bars represent the 95% highest posterior density (HPD) interval for the divergence times.

The BI analyses performed with 218 and 153 spp. to control for the bias of non-overlapping characters, and using 9 or 5 calibration points to control for less robust times of divergence, resulted in phylogenies that were consistent except for the placement of the *polychaeta* and *angor* groups (Fig. 1–3). The phylogeny obtained with the ML approach is also consistent in topology with the 218 (5 calibration points) and 153 spp. (Fig. 1). These results show that the presence of non-overlapping sequences is not a significant source of bias in the present study. However, the times of divergence estimated with 5 calibration points were significantly younger than those resulting from the analyses with 9 points (Fig. 1–3), which is likely the result of the removal of those at deeper nodes.

The crown age of the subgenus *Drosophila* is placed in the Eocene/Oligocene transition around 34.33 Mya (30.24–38.30 Mya 95% HPD) when all 9 calibration points are used, while it is placed in the Oligocene/Miocene transition, 23.79 Mya (19.24–28.83 Mya 95% HPD), when the calibration points used are reduced to 5 (Fig. 1 and 3). The phylogeny of the subgenus includes two clades corresponding to the *immigrans-tripunctata* [98% and 100% Bayesian Posterior Probability (BPP) in the BI with 9 and 5 calibrations, respectively; 60% bootstrap in the ML analysis] and the *virilis-repleta* (98% and 100% BPP; 55% bootstrap) radiations. The analysis using 9 calibration points places the crown ages of these lineages during the early Oligocene around 31 Mya (27.23–35.46 and 27.29–35.13 for the *immigrans-tripunctata* and *virilis-repleta*, respectively). When 5 calibration points were used the crown ages of these lineages were placed at 20 Mya (15.84–24.38) and 22.86 Mya (18.67–27.76) for the *immigrans-tripunctata* and *virilis-repleta* lineages, respectively.

The first lineage to diverge within the *virilis-repleta* radiation in the BI phylogeny with 218 spp. and 9 calibration points is the *polychaeta* species group, with a crown age of 26.63 Mya (22.12–30.93). However, in the ML and BI (218 spp. and 5 calibration points; 153 spp.) trees this lineage is placed as the sister group to the clade comprising the *annulimana*, *nannoptera*, *dreyfusi*, *canalinea*, *mesophragmatica* and *repleta* species groups, with bootstrap support <50% and 99% BPP. The crown age of the *polychaeta* species group estimated in this case was 18.42 Mya (14.56–22.81) The monophyly of the *repleta* radiation, which includes the *repleta*, *mesophragmatica* and *canalinea* species groups, is recovered although it is only well supported in the BI trees (85% to 91% BPP). Its sister clade is formed by the *nannoptera* and *annulimana* species groups. *D. camargoi*, member of the *dreyfusi* species group, is placed as sister species to the *nannoptera*, although this is not well supported by BPP or bootstrap. The crown age of the *repleta* radiation is estimated to be 24.36 Mya (21.04–27.78) or 18.31 Mya (14.61–22.36) with 9 or 5 calibration points, respectively, and it shared a last common ancestor with its sister species groups (*nannoptera* and *annulimana*) 27.76 Mya (24.34–31.50, 218 spp., 9 calibration points) or 19.78 Mya (15.83–24.12, 218 spp., 5 calibration points). The clade comprising the *virilis*, Hawaiian Drosophilidae, *robusta*, *melanica*, *quadrisetata* and *angor* species groups is recovered with different support (Fig. 1 and 3). This clade has an estimated crown age of 29.71 Mya (26.37–33.32) and 22.02 Mya with 9 and 5 calibration points, respectively. The first group to diverge in this clade is the *angor* group, with an estimated crown age of 24.91 Mya (21.23–28.48), but surprisingly it is not recovered as monophyletic in the BI analysis with 5 calibration points. Also, the *angor* species group is placed as the sister clade of the *polychaeta*-*repleta* lineage in the BI analysis with 153 spp. and 9 calibration points (Fig. 2). Monophyly of the Hawaiian Drosophilidae, *virilis*, *robusta*, *melanica* and *quadrisetata* species groups is well supported in the BI trees (100% and 89% BPP with 9 and 5 calibration nodes, respectively) although the bootstrap support is low in the ML analysis (56%). The crown age of this clade is 27.95 (24.68–31.41) Mya or 21.15 (16.79–25.46) Mya with 9 or 5 calibration nodes, respectively. The position of the Hawaiian *Drosophila* differs in the phylogenies, being monophyletic with *virilis* in the BI analysis of 218 spp. and 9 calibration points, but being recovered as the sister clade to the *virilis*, *robusta*, *melanica*, *quadrisetata* and *clefta* species groups in the other analyses (Fig. 2–3). The member of the *immigrans* clade *D. annulipes* is placed as sister species to the Hawaiian *Drosophila* with high support (Fig. 1 and 3). The *robusta* species group is recovered as polyphyletic in the present analysis and very closely related to the *melanica* and *quadrisetata* species groups.

The first species of the *immigrans-tripunctata* radiation to diverge is *D. quadrilineata*, a member of the *immigrans* group. The sister group is subdivided in two clades, one corresponding to the *immigrans* group and the second clade including the *tripunctata*, *pallidipennis*, *quinaria*, *cardini*, *guarani*, *testacea*, *macroptera*, *calloptera* and *funebris* species groups (Fig. 1–3). These two clades have crown ages corresponding to the Oligocene/Miocene transition or mid-Miocene depending on the analysis. Thus, the *tripunctata* and closely related groups have a crown age of 24.84 (21.69–28.04) Mya with 9 calibration points, or 17.48 (14.05–21.33) Mya with 5 calibration points. The *immigrans* species group (excluding *D. quadrilineata*) have crown ages of 22.95 (19.02–26.83) Mya (9 calibration points) or 15.57 (11.91–19.57) Mya (5 calibration points). The *tripunctata* is recovered as polyphyletic. The species *D. funebris* is also recovered as closely related to the *quinaria* and *macroptera* species groups, although its placement differs between the BI and ML trees.

### Patterns of Geographical Dispersal

**Figure 4:**
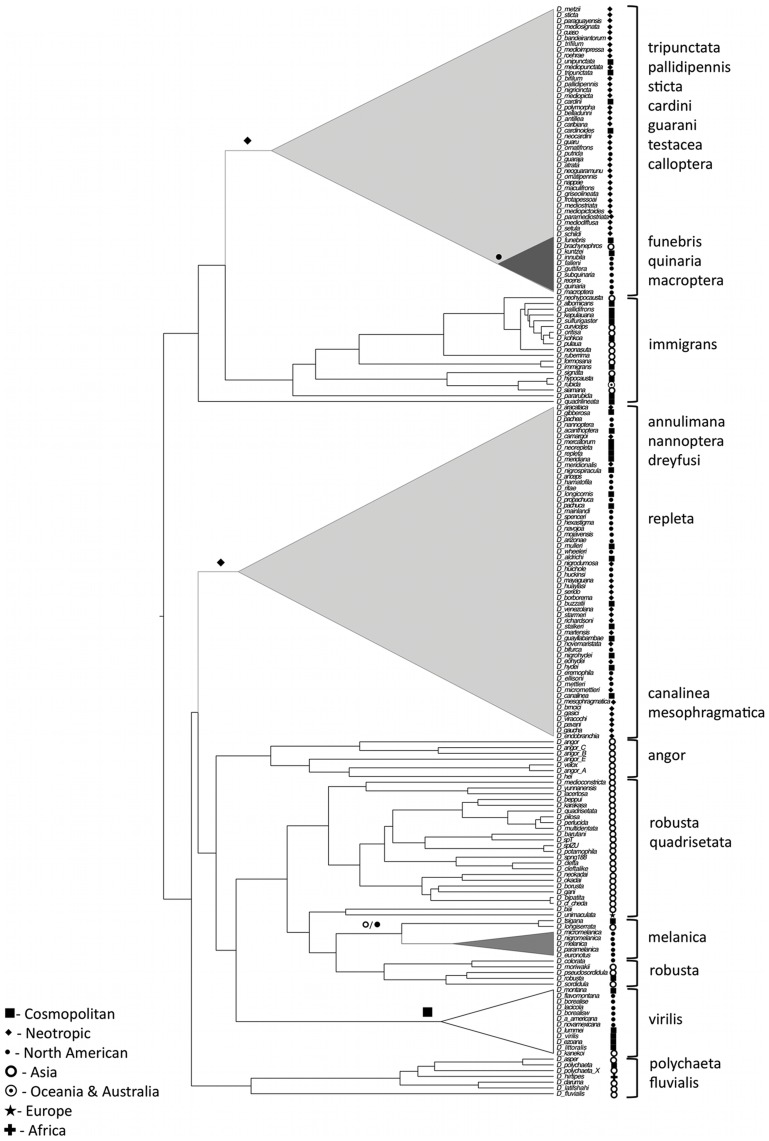
Ancestral reconstruction of geographic distribution and ecological niche. Trees showing the main dispersal events (A) and ecological shifts (B) inferred to have occurred during the evolution of the *Drosophila* subgenus. Clades involved are collapsed and the shift indicated with symbols above the branch.

Results obtained with the ML and Bayesian approaches are consistent (Fig. 4A and Table 2). Analyses placed the root of the *Drosophila* subgenus in Asia. The *immigrans-tripunctata* clade and the *immigrans* species group were inferred to have an Asiatic origin, while the lineage comprising the *tripunctata*, *pallidipennis*, *quinaria*, *cardini*, *guarani*, *testacea*, *macroptera*, *calloptera* and *funebris* species groups had a last common ancestor in the Neotropics. Within this clade, the last common ancestor of the cosmopolitan *D. funebris*, the North American *quinaria* species group (although it includes the Asiatic *D. brachynephros* and the cosmopolitan *D. kuntzei*) and *D. macroptera* was inferred to have a North American distribution, although this is better supported in the Bayesian analysis than in the ML analysis [marginal posterior probability (PP) = 0.95, proportional likelihood (PL) = 0.55]. The remaining species groups of this clade (*tripunctata*, *guarani*, *pallidipennis*, *calloptera*, *cardini* and *testacea*) all have a last common ancestor with an unequivocal Neotropical range.

**Table 2:** Ancestral biogeographic reconstructions obtained with Likelihood (ML) and Bayesian approaches.

	ML approach		Bayesian approach	
Node	region	PL	region	MPP
*Drosophila* subgenus	Asia	0.92	Asia	0.90
*immigrans-tripunctata*	Asia	0.85	Asia	0.80
*immigrans* a	Asia	0.93	Asia	0.92
(trip,pall,quin,card,gua,test,macrop,call,fun)	Neotropics	0.98	Neotropics	0.99
*quinaria*, *funebris*, *macroptera*	North America	0.55	North America	0.95
*virilis-repleta*	Asia	0.96	Asia	0.99
*polychaeta* + *D. fluvialis*	Asia	0.98	Asia	0.97
(vir, rob, mel, quad, ang)	Asia	0.99	Asia	0.99
*Virilis*	>1 region	0.85	>1 region	0.83
*robusta, melanica, quadrisetata*	Asia	0.99	Asia	0.99
*Melanica*	Asia	0.81	North America	0.63
*Quadrisetata*	Asia	0.99	Asia	0.99
*rpl, ann, nan, drey, cana, meso*	Neotropics	0.68	Neotropics	0.75
*repleta* radiation	Neotropics	0.72	Neotropics	0.67
*repleta*	Neotropics	0.69	>1 region	0.97

a excluding D. quadrilineata.

The *virilis-repleta* radiation is estimated to have an Asiatic origin. This is further supported by the fact that the first lineage to diverge, the *polychaeta*, includes species that are mainly Asiatic. Of the two main clades of the *virilis-repleta* radiation, the one comprising the *virilis*, *robusta*, *melanica*, *quadrisetata* and *angor* species groups had a last common ancestor in Asia. Within this Asiatic clade, two species groups have dispersed to other geographic regions. The ancestral range of the *virilis* species group is estimated as cosmopolitan, which is in agreement with the holarctic origin inferred in a recent analysis of the systematics of this group [29]. The ancestral species of the *melanica* species group has an equivocal distribution being inferred as Asiatic by the ML approach (PL = 0.81) and as North American with the Bayesian method (North America PP = 0.63 vs. Asia PP = 0.32). The Neotropical origin of the *repleta* radiation and closely related species groups is supported by both approaches (PL = 0.68 and PP = 0.75). Similarly, the ancestral species of the *annulimana*, *nannoptera* and *D. camargoi* lineage and the *repleta*, *mesophragmatica* and *canalinea* are also estimated to be Neotropical. Nevertheless, the *repleta* species group has an equivocal origin, as the ML analysis supports a Neotropical origin (PL = 0.69 vs. PL = 0.27 for a distribution across more than one region, namely the Neotropics and North America) and the Bayesian method inferred an ancestral distribution across more than one region (PP = 0.97 Neotropics and North American). A more detailed analysis of the biogeographic history of the *repleta* species group would need a better resolution of the phylogenetic relationships of the species it comprises.

### Evolution of Ecological Resource Use

Results of the ancestral reconstruction of the ecological resource use are shown in Fig. 4B and Table 3. Results indicate that the ancestor of the *Drosophila* subgenus was a generalist, although the support for this state is not strong in either of the two approaches (PL = 0.57 and PP = 0.55). The alternatives, however, show also low probabilities (the second most supported state is frugivore with a PL = 0.20 and PP = 0.27). The inferred ancestral state of the *immigrans* species group is also equivocal; the ML method results indicate that the ancestral species of the group was a generalist (PL = 0.51) or frugivorous (PL = 0.48), the Bayesian approach supports the frugivorous ancestral state with a posterior probability of 0.88 (the generalist state has a PP of 0.11). The majority of the species of this group included in the analyses are frugivorous, nevertheless, six species (*D. albomicans*, *D. oritisa, D. ruberrima, D. signata, D. immigrans* and *D. curviceps*) show a more generalist ecological usage having been caught in tree sap, fungi and fruit (table S2 and references included). This indicates that at least some species retained the ancestral character state of the *immigrans-tripunctata* lineage. Within the *immigrans-tripunctata* lineage the *quinaria* and *macroptera* groups are specialized in using fungi as resource [although some of the species of this group may be generalist such as *D. quinaria* [13]]. In our analyses, it is equivocal when these groups became mycophagous. While the ML reconstruction approach inferred that the ancestor of the *funebris*, *quinaria* and *macroptera* was a generalist with a proportional likelihood of 0.89, the Bayesian method resulted in a posterior probability of being mycophagous of 0.55 (0.44 for the generalist state). Although the species of the *funebris* group use fungi as resource, they can also use other resources [13]. The ML and Bayesian methods indicate that the ancestral species of the *quinaria* and *macroptera* was mycophagous (PL = 0.98 and PP = 0.99). It should be noted that some of the generalist species of the *immigrans* group also use fungi as ecological resource; therefore, we interpret this as evidence that the mycophagous state is not an apomorphy but that instead, species have specialized in fungi probably without losing the capacity to use other ecological resources.

**Table 3:** Ancestral reconstruction of the ecological resource used obtained with Likelihood (ML) and Bayesian approaches.

	ML approach		Bayesian approach	
Node	resource	PL	resource	MPP
*Drosophila* subgenus	unspecific	0.57	unspecific	0.55
*immigrans-tripunctata*	unspecific	0.80	unspecific	0.98
*immigrans* a	unspecific	0.51	frugivorous	0.88
(trip,pall,quin,card,gua,test,macrop,call,fun)	unspecific	0.99	unspecific	0.99
*quinaria*, *funebris*, *macroptera*	unspecific	0.89	mycophagous	0.55
*quinaria*, *macroptera*	mycophagous	0.98	mycophagous	0.99
*virilis-repleta*	unspecific	0.38	frugivorous	0.47
*polychaeta* b	frugivorous	0.95	frugivorous	0.99
(vir, rob, mel, quad)	sap feeders	0.90	sap feeders	0.99
*virilis*	sap feeders	0.99	sap feeders	0.99
*robusta, melanica, quadrisetata*	sap feeders	0.99	sap feeders	0.99
*melanica*	sap feeders	0.99	sap feeders	0.99
(rpl, ann, nan, drey, cana, meso)	cactophilic	0.55	cactophilic	0.85
*repleta* radiation	cactophilic	0.70	cactophilic	0.68
*repleta*	cactophilic	0.91	cactophilic/unspecific+cactophilic	0.49/0.49

a excluding D. quadrilineata.

b not including the unclassified species D. fluvialis.

The ecological resource of the last common ancestor of the *virilis-repleta* radiation is equivocal. The ML approach reconstructed the node as generalist (PL = 0.38; the alternative states frugivore and sap feeder had PL = 0.24), while the Bayesian method estimated the ancestor to be frugivorous with a posterior probability of 0.47 (sap feeder PP = 0.25, cactophilic PP = 0.16 and generalist PP = 0.10). The *polychaeta* group includes frugivorous species, and the ancestral state of this lineage is here inferred to be frugivorous by both methods. The remaining lineages of the *virilis-repleta* radiation can be classified depending on the ecological resource they use. The sap feeder species groups, the *virilis*, *robusta*, *melanica* and *quadrisetata* forms one of the two monophyletic lineages of the radiation; the second clade includes the *repleta* radiation and closely related species groups, which are predominantly cactophilic. Nevertheless, within this second clade there are species that are frugivorous such as the *annulimana* species group, or that are able to utilize other plant parts besides cacti, such as some species of the *repleta* group ([52], and references therein). However, it is inferred that the ability to use cacti as an ecological resource first appeared in the last common ancestor of these groups (PL = 0.55 and PP = 0.85).

### Diversification Analysis

Results of the tests obtained with the phylogenies from both BI inferences were consistent (Table 4 and Table S3). Thus, henceforth it is reported only the results from the analyses run with the 218 spp. and 9 calibration points.

**Table 4:** Results of fitting diversification models to the *Drosophila* subgenus (A), the *tripunctata* and closely related species groups (B), and the *repleta* and closely related species groups (C).

A)	pureBirth	BD	DDL	DDX	yule2rate	yule3rate
*Parameters*	r1 = 0.087	r1 = 0.087a = 0	r1 = 0.126k = 436.00	r1 = 0.630x = 0.430	r1 = 0.161r2 = 0.068st = 13.34	r1 = 0.161r2 = 0.089r3 = 0.057st1 = 13.34st2 = 7.42
*Ln(L)*	211.705	211.705	223.905	227.668	229.101	232.127
*AIC*	−421.410	−419.410	−443.810	−451.337	−452.203	−454.254
*ΔAIC*	32.844	34.844	10.444	2.916	2.051	0
P = 0.9634						
**B)**	**pureBirth**	**BD**	**DDL**	**DDX**	**yule2rate**	**yule3rate**
*Parameters*	r1 = 0.069	r1 = 0.069a = 0	r1 = 0.255k = 51.083	r1 = 1.796x = 0.975	r1 = 0.181r2 = 0.034st = 12.44	r1 = 0.325r2 = 0.110r3 = 0.030st1 = 17.61st2 = 10.85
*Ln(L)*	−31.142	−31.142	−12.964	−16.367	−15.299	−11.293
*AIC*	64.285	66.285	29.927	36.734	36.599	32.585
*ΔAIC*	34.358	36.358	0	6.807	6.672	2.658
P = 0.0000						
**C)**	**pureBirth**	**BD**	**DDL**	**DDX**	**yule2rate**	**yule3rate**
*Parameters*	r1 = 0.090	r1 = 0.090a = 0	r1 = 0.158k = 82.055	r1 = 0.434x = 0.475	r1 = 0.149r2 = 0.065st = 10.38	r1 = 0.149r2 = 0.058r3 = 0.141st1 = 10.38st2 = 0.63
*Ln(L)*	−14.003	−14.003	−10.824	−10.251	−9.269	−7.943
*AIC*	30.006	32.006	25.647	24.501	24.538	25.886
*ΔAIC*	5.504	7.504	1.146	0	0.037	1.385
P = 0.2951						

The phylogeny used was that obtained using the 218 species and 9 calibration points. P indicates the significance of the *ΔAIC* between the rate-constant and rate-variable models. (BD – Birth-Death model; DDL – Density-dependent logarithmic model; DDX – Density-dependent exponential model).

The estimated mean γ value for the posterior distribution of trees of the *Drosophila* subgenus is −5.184201 (−4. 105418 to −6.363904), indicative of a deceleration of the speciation rate. However, results of the MCCR test (total number of species 1506, missing species 1288) indicate that the negative value obtained from our distribution of trees is not significant when compared with the null distribution (critical γ value −9.019494, P = 1). The BDL analysis resulted in a better fit of the rate-variable (RV) models than the rate-constant (RC) models, and found the yule3rate model as being the best RC model (Table 4A). Nevertheless, the improvement in AIC (ΔAIC) of the RV models was not significant when compared to that observed in a null sample of 5000 simulated trees (P = 0.9634). This result is congruent with the CR test and both indicate that a constant mode of evolution of the *Drosophila* subgenus cannot be rejected.

In order to analyse the effect that the dispersal to the New World may have had in the diversification pattern of particular lineages of the subgenus *Drosophila*, we analysed the clade including the *tripunctata*, *pallidipennis*, *cardini*, *guarani*, *testacea*, *calloptera*, *funebris*, *quinaria*, *sticta* and *macroptera* species groups. These species groups have a last common ancestor with an inferred Neotropical distribution. The estimated mean γ value for the posterior distribution of 1000 trees is −5.359199 (−4.558396 to −6.304164). The MCCR test (assuming a total number of species of 170 and 120 missing) indicated that the value obtained for the posterior distribution trees is significant (critical γ value −3.789056, P<0.0006). Furthermore, the BDL analysis found the RV models to fit better the data than the RC. The logarithmic density-dependent model (DDL) model showed the lowest AIC value (Table 4B). This ΔAIC of the RV models was significant when compared to a null sample obtained from 5000 simulated trees (P = 0). Nevertheless, the γ value loses its significance at low levels of non-random sampling (α = 0.55; P>0.05). This indicates that a type-I error is likely with a little degree of sampling bias and therefore, the significance of the signal of a deceleration in the rate of diversification with time could be the result of a bias due to non-random sampling.

Similarly to the *tripunctata* and closely related species groups, the *repleta*, *annulimana*, *nannoptera*, *dreyfusi*, *canalinea* and *mesophragmatica* species groups had a last common ancestor with a New World distribution. Furthermore, the ability to use cacti as ecological resource was estimated to have evolved in this last common ancestor. The mean γ-statistic value estimated for 1000 trees of the posterior distribution obtained with BEAST is −2.0340890 (interval from −0.897719 to −3.039011), indicating a decrease in the lineage accumulation as time proceeds. Nevertheless, this value is not significant as indicated by the MCCR test (total number of species 158, missing 100) (critical γ value = −3.505972; P = 0.5087). Furthermore, although the BDL analysis found a RV model (DDX) to have the best fit to our data (Table 4C), this improvement was not significant when compared to a null sample of simulated trees (P = 0.2951).

The effect that adaptation to cacti may have had in the speciation rate of the clade including the *repleta* and closely related groups has been also tested using BiSSE. Results show no statistically significant difference in the speciation rates between the cactophilic clades (λ1 = 0.0856) and the non-cactophilic (λ0 = 0.0846) (P>0.05).

**Figure 5:**
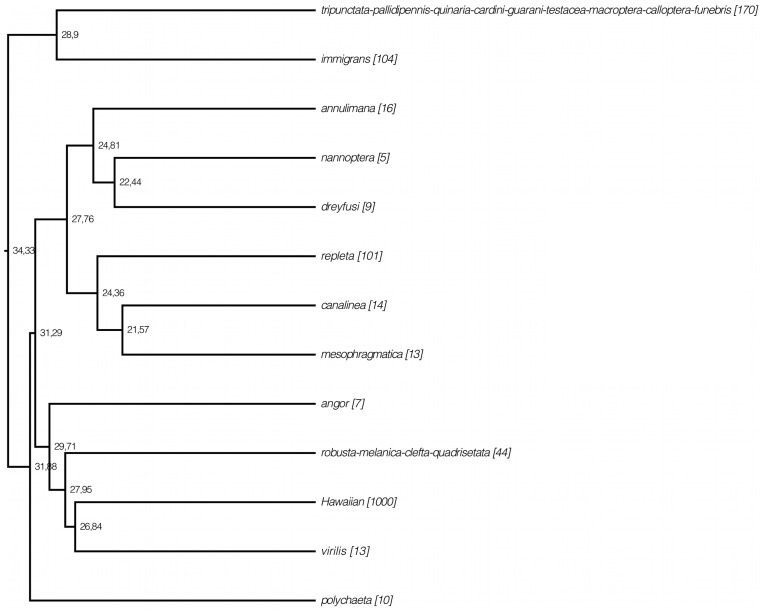
Backbone topologies used in the MEDUSA analyses. A) phylogeny backbone from analysis with 218 species and 9 calibration points; B) phylogeny backbone from analysis with 218 species and 5 calibration points. Tip names refer to the species groups (those polyphyletic were clustered into a single clade) and numbers in brackets refer to the species richness of the tip. Numbers on nodes indicate divergence times.

The comparative method MEDUSA was used to detect clades with increased rates of diversification within the subgenus. Given that poorly resolved lineages can bias the results of this method [53], two backbone trees were used to test for uncertainty bias in the topology and divergence times (Fig. 5). Results are similar and indicate that the net rate of diversification is significantly greater in the Hawaiian Drosophilidae (r = 0.20) than in the other groups of the subgenus (r = 0.14), but among the non-Hawaiian lineages there are no differences in the rate of diversification. This method assumes a constant rate of diversification through time, which is met, as the analyses above indicate no departure from the CR models.

## Discussion

### Phylogenenetic Inferences in the Subgenus *Drosophila*

The phylogenies obtained in the present analysis are in general agreement with previous studies including some of the groups here analysed [12], [15], [17], [18], [54]. Thus, two major clades are identified, one corresponding to the *immigrans-tripunctata* radiation and the other one corresponding to the *virilis-repleta* radiation, that also includes the Hawaiian Drosophilidae [15], [17], [18]. Also in agreement with the previous studies is the paraphyly of the *tripunctata*, *immigrans*, *guarani*, *calloptera* and *robusta* species groups [54], [55], [56], [57]. Within the *virilis-repleta* radiation, the position of the *polychaeta* group is not well resolved. In the BI tree (218 spp. and 9 calibration points) the *polychaeta* lineage is the sister group to all other species groups, a topology consistent with previous studies [15], [54], [58]. On the other hand, the remaining BI and ML analyses place the *polychaeta* as the sister group to the clade comprising the *repleta*, *annulimana*, *nannoptera*, *dreyfusi*, *canalinea* and *mesophragmatica*, a relationship observed previously with mitochondrial markers [54]. The sister relationship between the Hawaiian *Drosophila* and the *virilis* group recovered in the BI analysis (218 spp., 9 calibration points) is novel, nevertheless this is not supported by the BPP. On the other hand, the other BI and ML analyses placed the Hawaiian *Drosophila* as the sister group of the *virilis-robusta-melanica-quadrisetata* clade, a relationship that has been observed in other studies [18]. Another alternative hypothesis place the Hawaiian Drosophilidae as the sister lineage of the *virilis-repleta* [15], [17] and cannot be ruled out. The placement in the present analysis of *D. annulipes*, a member of the *immigrans* species group, as the sister species to the Hawaiian *Drosophila* is similar to that obtained by Katoh *et al*. [57].

Within the *immigrans-tripunctata* lineage, two clades are recovered in the present study, one corresponding to the *immigrans* species group and a second one comprising the *tripunctata*, *pallidipennis*, *quinaria*, *cardini*, *guarani*, *testacea*, *macroptera*, *calloptera* and *funebris*. This is consistent with previous studies [12], [17], [18], [55], [56]. The sister species to these two clades is a member of the *immigrans* species group, *D. quadrilineata*, rendering this group paraphyletic; however, this position has been reported in a previous study [57]. The polyphyly of the *tripunctata* species group here observed is in agreement with other recent study of the group [55]. The *D. funebris* is placed in the present study together with the *quinaria* and *macroptera* species groups. Although the *funebris* species group was considered by Throckmorton [11] to be the sister group of all other groups of the subgenus *Drosophila*, previous molecular analyses have also placed this group within the *immigrans-tripunctata* radiation [15], [16], [56].

Few previous studies have attempted to date the phylogeny of the subgenus *Drosophila*. Throckmorton [11] based on biogeographic information proposed an evolutionary history for the Drosophilidae, although the divergence times of lineages are only vaguely specified. Other studies have dated the divergence of some of the groups using immunological and DNA sequence data [37], [59], [60]. Furthermore, two studies have attempted to calibrate the molecular clock of *Drosophila* using mutation rates as an approximation of substitution rates [61], [62]. All these studies have used few species and have resulted in contrasting times of speciation. These differing times of divergence observed among studies reflect the uncertainties of the assumptions of the different methods used. For example, different models of emergence and colonisation of the Hawaiian islands result in contrasting times of speciation, or the use of mutation rates to estimate times of divergence also rely on the use of appropriate generation times [62]. The use of reliable points of calibration will be of relevance in obtaining good estimates of species divergence. It is also expected that the more points are used, the more reliable the estimation will be. The present study is the first one to estimate times of divergence using a relaxed molecular clock and a large number of species. The scarcity of fossil data poses a challenge towards estimating the origin and evolutionary history of this group of organisms. Few fossils belonging to Drosophilids have been described to date from samples of Dominican Republic amber, estimated to have been deposited during the early Miocene (∼23 Mya) [63]. One of the few is a member of the genus *Scaptomyza* [63], which is the sister group of the Hawaiian *Drosophila* and thus within the *virilis*-*repleta* lineage. Other two extinct species from Dominican Republic amber, *D. poinari* and *D. succini*, have been described samples as belonging to the genus *Drosophila* (and sharing some morphological features with members of the subgenus *Drosophila*) [63]. These fossils suggest that by the Oligocene/Miocene transition some of the lineages of the subgenus *Drosophila* were already diverging. This is more compatible with the crown age of the subgenus *Drosophila* being 34.33 Mya estimated with 9 calibration points than the 23.79 Mya estimated with 5 calibration points.

According to the results from the analysis including more calibrations, the divergence between the *Drosophila* and the *Sophophora* (outgroup) subgenera occurred around 36 Mya, which is a much younger estimate than the 61–65 Mya estimated using immunological distances [59] and synonymous mutation-based molecular clock [37], but is similar to the 39 Mya estimated using the *Adh* gene [60] and the 32 (25–40) Mya estimated using the mutation rate as a proxy for substitution rate [62]. The crown age for the *Drosophila* subgenus (and, therefore, the divergence of the two major lineages, the *immigrans-tripunctata* and *virilis-repleta*), is in the present study placed in the late Eocene, approximately 34 Mya, which is similar to the ∼33 Mya divergence estimate between the *D. immigrans* and *D. repleta* groups by Russo et al. [60]. This is also consistent with the Oligocene date proposed by Throckmorton [11]. Beverly and Wilson [59] estimated the divergence of the *repleta* and *robusta* groups to be 35 Mya, an age that is consistent with the ∼31 Mya our results indicate. The divergence between the *mettleri* and *mulleri* subgroups of the *repleta* were estimated by Russo et al. [60] to be of ∼16 Mya, while the estimate in the present analysis is of ∼21 Mya. Oliveira et al. [64], using as calibration points those divergence times of Russo et al. [60] have also estimated a crown age for the *repleta* species group of ∼16 Mya, an estimate more similar to our results using 5 calibration points. Nevertheless, the phylogenetic resolution within some groups is not well supported. Results here presented indicate that the major lineages within the two radiations appeared between the late Oligocene and the first age of the early Miocene (28.5–20.5 Mya), and by the middle Miocene epoch most of the species groups had already diverged. This period of time corresponds with a long-term trend of climate warming that started from 26–27 Mya and lasted until the middle Miocene (15 Mya), with the exception of brief periods of glaciation approximately 23 Mya [65]. These climatic conditions are likely to have influenced speciation in *Drosophila* as well as in other biota.

### Patterns of Evolution in the *Drosophila* Subgenus

A commonly observed pattern of diversification is that lineage diversification rates decline through time (e.g. [7], [66]). This density-dependent trend can be explained by an early greater opportunity for occupying new ecological niches where the competition pressure is reduced and allows for a rapid diversification rate [5], [6], [7]. This is followed by a decrease in the speciation rate as the niche becomes saturated and the competition for ecological space increases. Some authors have suggested that the diversification of the main lineages of the *Drosophila* subgenus occurred rapidly early in the evolution of the group, remaining stable for a long time until the present [12], [67]. Furthermore, the subgenus contains a large number of species that show a considerable diversity in geographic distribution and use a significant variety of ecological resources. These features are suggestive of an adaptive radiation. However, results do not support the hypothesis that the *Drosophila* subgenus is an adaptive radiation.

Dispersal into new areas and evolution of characters that allow the use of new resources, often result in an ecological opportunity that leads to adaptive radiations [2], [5]. Dispersal of lineages of the *Drosophila* subgenus to the New World has occurred at least twice independently and at similar times; both, the ancestors of the *tripunctata* and closely related species groups, and the *repleta* and closely related species groups had a Neotropical distribution. These results support the Neotropical origin of these lineages proposed by Throckmorton [11]. In contrast with the origin of *repleta* and closely related species groups, our analyses have not been able to resolve the ancestral distribution of the *repleta* species group. Similarly, a recent analysis of the ancestral geographic distribution of the *repleta* species group was not able to place the origin of this group in either North America or South America, and the authors suggest that the biogeographic history of this group is marked by a repeated exchange of fauna between these subcontinents [64]. Our results would support the hypothesis of a close relationship between the *repleta* subgroups of North and South America. Also, several resource shifts are here inferred to have occurred from the generalist ancestral state of the subgenus. Thus, the ancestor of the *repleta* and closely related species groups shifted to a cactophilic state, also observed in a recent study of this group [64], that of the *immigrans* and the *polychaeta* groups became frugivorous, and the *quinaria* and *macroptera* shared a common ancestor that was mycophagous. Within the *repleta* species group, shifts from *Opuntia* to columnar cacti species have occurred several times independently [64]. Despite these dispersal events and ecological shifts, we are not able to detect in any of the clades tested a pattern of speciation through time consistent with a density-dependent model, which would be indicative of an adaptive radiation [7], [8]. Furthermore, apart from the Hawaiian Drosophilidae, there is no evidence for higher rates of diversification among the other species groups of the subgenus, further supporting the lack of influence in the net diversification rate of the ecological shifts or geographic dispersals.

Surprisingly, the lineage of cactophilic species groups does not show any departure from a constant rate of speciation. Despite colonising the Neotropics and acquiring the capacity to exploit cacti as ecological resource, no signature of adaptive radiation has been detected and there is no difference in the diversification rate between cactophilic and noncactophilic lineages. These results contrast with the recent suggestion of a rapid radiation of the *repleta* species group along its cacti hosts [64]. However, Oliveira et al. do not test for an increase in the rates of speciation in this group of species [64]. In contrast we have specifically tested the hypothesis of an increased rate of speciation in this lineage as a result of becoming cactophilic, and were not able to reject the constant rate model. Thus, there is no support for a rapid radiation of the *repleta* species group. There is evidence for genetically differentiated host-races in some of the cactophilic species, indicating the relevance of cactus species use in the evolution of this group. However, this differentiation sometimes reflects geographic separation and races show no reproductive isolation [38], [68], [69], [70], [71]. Thus, adaptation to different cacti species does not necessarily have to be associated to an increase in the rate of speciation, even though it might be relevant for the evolution of the clade.

Despite not having found evidence for adaptive radiation in the subgenus *Drosophila* in relation to ecological opportunity as a result of colonization of new geographic regions or new ecological resources, it is still possible that other intrinsic characteristics could have resulted in an increase in the speciation rate of other lineages than the ones explored here. Indeed, a recent study in cichlids shows that it is a combination of intrinsic characteristics and extrinsic factors that best explains the propensity to radiate of some lineages [72]. In particular, this study finds that sexual dichromatism, a better proxy than species mating system for sexual selection intensity, in combination with ecological opportunity (lake depth) influence the radiation pattern of cichlids [72]. Thus, the intensity of sexual selection in species of *Drosophila* could be a good candidate for future studies of diversification patterns.

### Conclusion

Our results show that the proposed *Drosophila* taxonomic radiations do not correspond to adaptive radiations. Furthermore, none of the ecological resource shifts or the geographic dispersal events observed in the phylogeny of the *Drosophila* subgenus can be unequivocally linked to an adaptive radiation of the clade. In particular, the evolution of cactophily should not be invoked as a general explanation for the diversity of the *repleta* group. Results lend support towards the idea that in some groups, the pace of diversification can be more limited by the rate of speciation (the time it takes to achieve reproductive isolation) than by the evolution of new traits or colonisation of new regions, and reproductive isolation may be a prior requisite for adaptive divergence to occur [6], [73].

## Supporting Information

Table S1 (XLSX)

Table S2 (XLSX)

Table S3 (DOCX)
