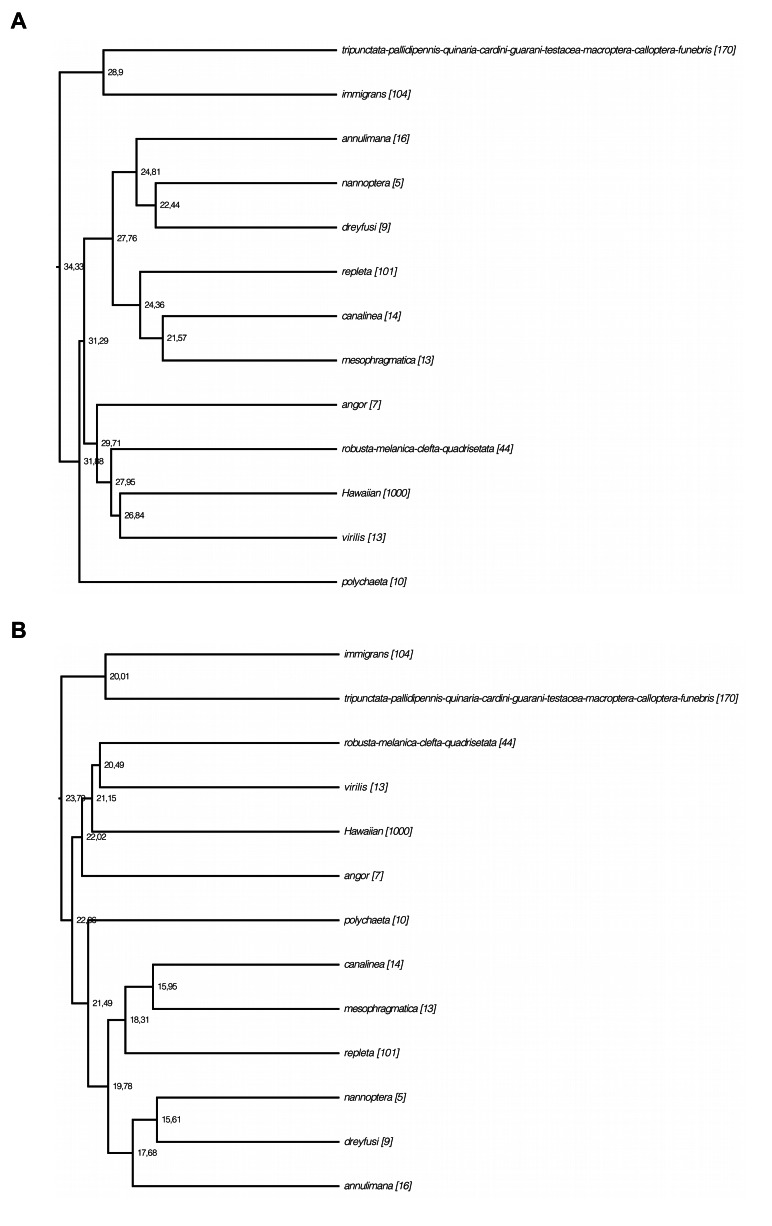# Correction: Phylogenetic Patterns of Geographical and Ecological Diversification in the Subgenus *Drosophila*

**DOI:** 10.1371/annotation/28ac6052-4f87-4b88-a817-0cd5743e83d6

**Published:** 2013-05-14

**Authors:** Ramiro Morales-Hojas, Jorge Vieira

The versions of Figures 1, 4, and 5 are incomplete. The complete, correct versions of these figures can be found here:

Figure 1: 

**Figure:**
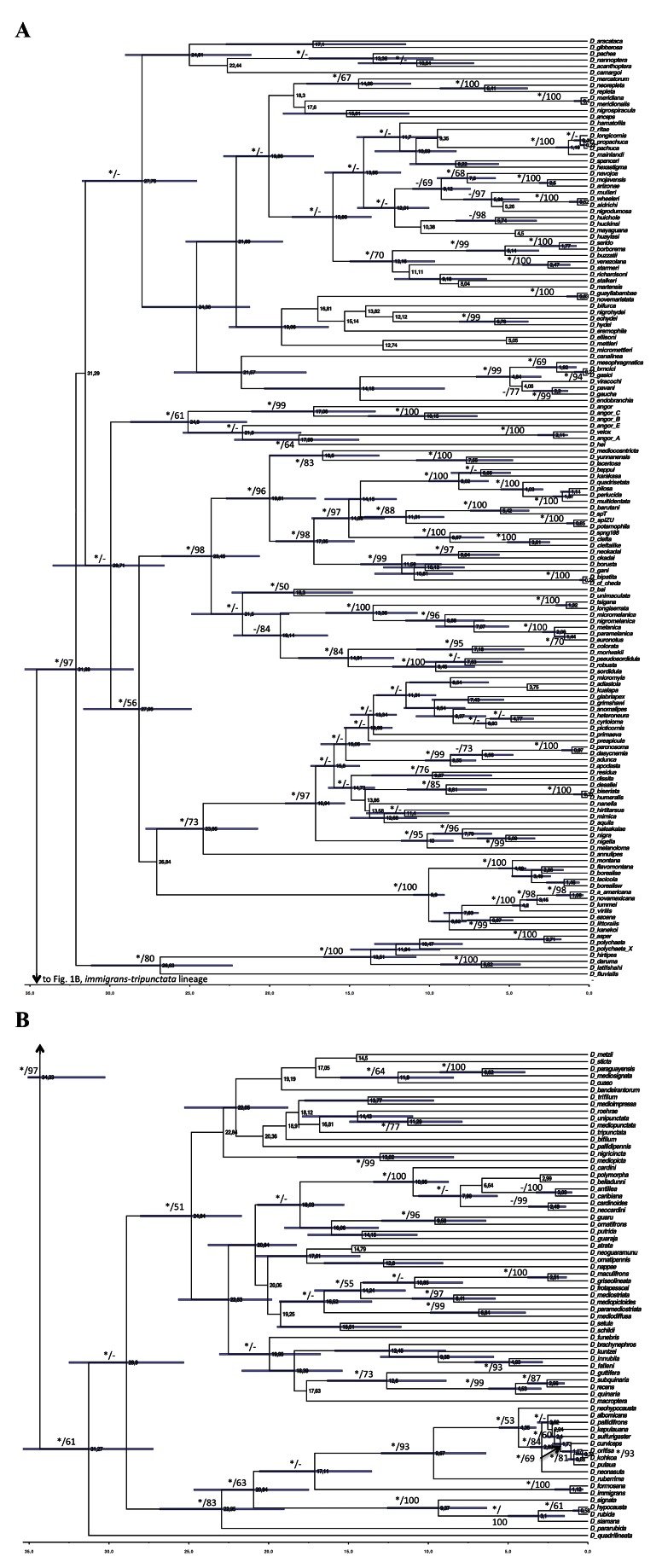


Figure 4: 

**Figure:**
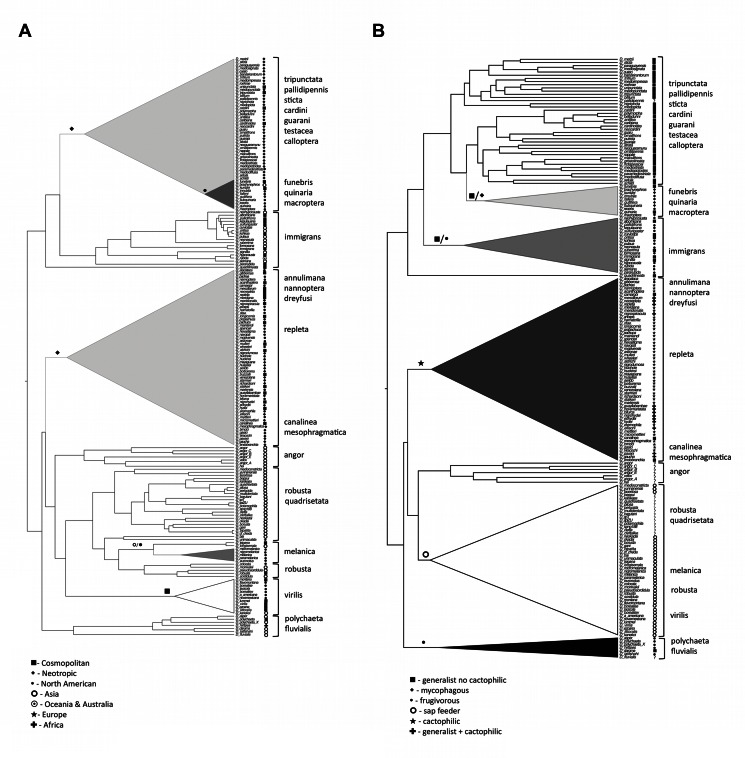


Figure 5: 

**Figure:**