# Supplementary material for: Phylogenetic Patterns of Geographical and Ecological Diversification in the Subgenus Drosophila
Source: PLoS One. 2012 Nov 12;7(11):e49552. doi: 10.1371/journal.pone.0049552 (PMC3495880; doi:10.1371/journal.pone.0049552)
Supplement: Table S3 — (DOCX) [file pone.0049552.s003.docx]

**Table 3 Supplemental.** Results of diversification tests using the phylogeny obtained with 153 species and 5 calibration points. *Drosophila* subgenus (A), *tripunctata* and closely related species groups (B), and *repleta* and closely related species groups (C). γ indicates the observed value of the posterior distribution of 1000 trees; critical-γ and associated P value indicate the value corrected for incomplete sampling and the significance of the observed γ value. NRS shows the results for the non-random sampling bias, with α being the scaling parameter value at which the MCCR test loses significance. Tables show the results of fitting diversification models to the different clades; P values below indicate the significance of the *ΔAIC* between the rate-constant and rate-variable models. (BD – Birth-Death model; DDL – Density-dependent logarithmic model; DDX – Density-dependent exponential model).

A)

γ = -4.766745; critical-γ = -8.735033 (P = 1)

|  | **pureBirth** | **BD** | **DDL** | **DDX** | **yule2rate** | **yule3rate** |
| --- | --- | --- | --- | --- | --- | --- |
| *Parameters* | r1 = 0.115 | r1 = 0.115  a = 0 | r1 = 0.224  k = 195.612 | r1=0.895  x = 0.483 | r1 = 0.170  r2 = 0.067  st = 5.649 | r1 = 0.266  r2 = 0.146  r3 = 0.067  st1=13.54  st2 = 5.64 |
| *Ln(L)* | 137.216 | 137.216 | 150.698 | 150.377 | 152.320 | 155.955 |
| *AIC* | -272.433 | -270.433 | -297.397 | -296.754 | -298.641 | -301.909 |
| *ΔAIC* | 29.476 | 31.476 | 4.512 | 5.155 | 3.268 | 0 |

P = 0.998

B)

γ = -4.582863; critical-γ = -3.577438 (P = 0.002). NRS: α = 0.6, P = 0.0556

|  | **pureBirth** | **BD** | **DDL** | **DDX** | **yule2rate** | **yule3rate** |
| --- | --- | --- | --- | --- | --- | --- |
| *Parameters* | r1 = 0.073 | r1 = 0.073  a = 0 | r1 = 0.702  k = 16.170 | r1 = 1.613  x = 1.298 | r1 = 0.209  r2 = 0.014  st = 8.562 | r1 = 0.328  r2 = 0.117  r3 = 0.007  st1 = 11.07  st2 = 8.40 |
| *Ln(L)* | -23.523 | -23.523 | -7.433 | -16.959 | -13.184 | -8.970 |
| *AIC* | 49.046 | 51.046 | 18.867 | 37.918 | 32.369 | 27.939 |
| *ΔAIC* | 30.179 | 32.179 | 0 | 19.051 | 13.502 | 9.072 |

P = 0.002

C)

γ = -2.420868; critical-γ = -3.427852 (P = 0.353)

|  | **pureBirth** | **BD** | **DDL** | **DDX** | **yule2rate** | **yule3rate** |
| --- | --- | --- | --- | --- | --- | --- |
| *Parameters* | r1 = 0.108 | r1 = 0.108  a = 0 | r1 = 0.224  k = 50.327 | r1 = 0.848  x = 0.685 | r1 = 0.167  r2 = 0.063  st = 6.19 | r1 = 0.313  r2 = 0.34  r3 = 0.063  st1 = 13.54  st2 = 6.19 |
| *Ln(L)* | -15.325 | -15.325 | -11.407 | -10.664 | -10.942 | -9.061 |
| *AIC* | 32.650 | 34.651 | 26.814 | 25.328 | 27.884 | 28.121 |
| *ΔAIC* | 7.322 | 9.323 | 1.486 | 0 | 2.556 | 2.793 |

P = 0.421
